# Monte Carlo Modeling of Photon Propagation Reveals Highly Scattering Coral Tissue

**DOI:** 10.3389/fpls.2016.01404

**Published:** 2016-09-21

**Authors:** Daniel Wangpraseurt, Steven L. Jacques, Tracy Petrie, Michael Kühl

**Affiliations:** ^1^Plant Functional Biology and Climate Change Cluster, University of Technology Sydney, SydneyNSW, Australia; ^2^Marine Biological Section, Department of Biology, University of CopenhagenHelsingør, Denmark; ^3^Department of Biomedical Engineering, Oregon Health & Science University, PortlandOR, USA

**Keywords:** coral tissue, coral optics, Monte Carlo simulation, photosynthesis, symbiosis, microorganisms, algae, biophysics

## Abstract

Corals are very efficient at using solar radiation, with photosynthetic quantum efficiencies approaching theoretical limits. Here, we investigated potential mechanisms underlying such outstanding photosynthetic performance through extracting inherent optical properties of the living coral tissue and skeleton in a massive faviid coral. Using Monte Carlo simulations developed for medical tissue optics it is shown that for the investigated faviid coral, the coral tissue was a strongly light scattering matrix with a reduced scattering coefficient of μ_s_’ = 10 cm^-1^ (at 636 nm). In contrast, the scattering coefficient of the coral skeleton was μ_s_’ = 3.4 cm^-1^, which facilitated the efficient propagation of light to otherwise shaded coral tissue layers, thus supporting photosynthesis in lower tissues. Our study provides a quantification of coral tissue optical properties in a massive faviid coral and suggests a novel light harvesting strategy, where tissue and skeletal optics act in concert to optimize the illumination of the photosynthesizing algal symbionts embedded within the living coral tissue.

## Introduction

Corals provide the building blocks of the most productive and diverse marine ecosystem, the coral reef (Hatcher, 1990); arguably one of the most spectacular manifestations of life on Earth. Reef building corals are invertebrates belonging to the family *Cnidaria*, akin to jellyfish and anemones, but in contrast to those soft-bodied animals, scleractinia corals secrete calcium carbonate and form an intricate skeleton matrix that supports the animal tissue. The living tissue is of fairly complex structure, containing various cell layers that, e.g., harbor a simple nervous system, muscle cells, and harpoon-like cells, known as cnidocytes, which are used for capturing their planktonic prey (Tardent, 1995). Although symbiont-bearing corals partly rely on heterotrophic energy via particle and prey capture, most (>95%) of their energy demand is covered by endosymbiotic microscopic dinoflagellate algae of the genus *Symbiodinum* (Falkowski et al., 1984) that carry out photosynthesis within the gastrodermis of the coral tissue. Symbiont photosynthesis excretes O_2_ and carbohydrates supporting coral host metabolism and calcification, while the coral host provides a protected environment and metabolic waste products such as inorganic carbon and nutrients that fuel symbiont photosynthesis (Muscatine et al., 1981).

The role of solar radiation in sustaining the successful photosymbiosis in corals has been a research focus for decades (Dubinsky et al., 1984; Falkowski et al., 1990; Dubinsky and Falkowski, 2011). The coral host must ensure sufficient light exposure of its microalgal symbionts and *Symbiodinium* photosynthesis *in hospite* is apparently highly efficient (Stambler and Dubinsky, 2005). Recent studies showed that the local quantum efficiency of *Symbiodinium* in coral tissue is close to the theoretical maximum of 0.125, i.e., eight photons per O_2_ molecule evolved (Brodersen et al., 2014). Photosynthetic efficiencies of planktonic free-living *Symbiodinium* are much lower than *in hospite* (Enriquez et al., 2005; Stambler and Dubinsky, 2005; Rodriguez-Roman et al., 2006; Brodersen et al., 2014). However, the coral symbiosis is, at the same time, highly susceptible to excess irradiance, especially in shallow reef areas during periods of elevated water temperatures (Glynn, 1996; Wangpraseurt et al., 2014b). The combination of excess irradiance and elevated temperature readily leads to the impairment of the photosynthetic machinery of the algae, i.e., chronic photoinhibition, which can trigger symbiont expulsion and the visible paling of the corals known as coral bleaching (Iglesias-Prieto et al., 1992; Brown, 1997; Jones et al., 1998). It is thus of great interest to resolve the mechanisms by which corals modulate their radiative exposure.

The optical properties of corals have been suggested to play a key role in determining both light stress susceptibility and photosynthetic efficiency (Falkowski et al., 1990; Enriquez et al., 2005; Stambler and Dubinsky, 2005; Kahng et al., 2012; Brodersen et al., 2014; Wangpraseurt et al., 2014a). Until now, there has only been a limited number of studies on coral optics and most studies have characterized the optics of the coral skeleton (Enriquez et al., 2005). It was shown that the skeleton of the coral *Porites branneri* was diffusely backscattering and that such backscattered light can enhance the local irradiance exposure of *Symbiodinium* (Enriquez et al., 2005). Further studies on coral skeleton optics have highlighted the role of skeleton scattering in coral bleaching susceptibility. Teran et al. (2010) developed a Monte Carlo simulation to show that the *in vivo* light field of *Symbiodinium* increases exponentially when a coral is bleaching, i.e., expelling the light absorbing elements. Marcelino et al. (2013) showed that there is a great variability in the magnitude of skeleton scattering and that there are species dependent differences in skeleton scattering, that partly correlate with coral bleaching susceptibility. While these studies provided insight into the light scattering properties of coral skeletons, much less is known about the optical properties of intact corals and specifically the optical properties of coral tissues.

Studies that investigated the scattering properties of coral skeletons have hitherto assumed that tissue scattering is negligible and that the refractive index of coral tissues is similar to water (Teran et al., 2010). Based on such assumptions, light is primarily absorbed in the tissue and backscattered by the skeleton leading to a fairly diffuse and homogenous light field within the tissue. Although the scattering properties of coral tissues have remained unknown, it is possible that skeleton scattering dominates the optical properties of intact corals if the tissue is thin and structurally simple, e.g., in thin-tissued Acroporid corals. However, there is a great range of corals with highly developed fleshy tissues. Such fleshy corals have tissue thicknesses up to several millimeters, a simple muscular system that facilitates tissue movement and the tissue can also harbor green-fluorescent protein-like pigment granules, which have been shown to have strong light scattering and reflective properties (Salih et al., 2000; Lyndby et al., 2016).

Recent studies on the optical properties of such fleshy coral tissues have shown that the tissue can transfer light laterally (Wangpraseurt et al., 2014a). Light microsensors were used to map the distribution of laser light incident on an intact coral. The results suggested that coral tissue was able to scatter light and that such tissue light scattering was found to affect coral photosynthesis at remote areas from the incident light beam (Wangpraseurt et al., 2014a). However, the details of light propagation through corals and the interaction between tissue and skeleton are still unclear, because the inherent optical properties of coral tissues that modulate tissue-light interactions remain unknown. The inherent optical properties quantify the probability of light absorption (absorption coefficient, μ_a_ [cm^-1^]), light scattering (scattering coefficient μ_s_ [cm^-1^]) and the direction of light scattering (the scattering phase function, which is often described by the anisotropy of scattering, *g* [dimensionless]). Another useful parameter is the reduced scattering coefficient μ_s_’ which is used to describe light scattering in the diffusional regime, i.e., where significant scattering events have occurred so that light moves by diffusion down photon concentration gradients (Jacques, 2013). Mathematically, μ_s_’ is defined as μ_s_’ = μ_s_ (1-*g*). Knowledge on spatially resolved coral skeleton and tissue optical properties would provide a mechanistic understanding of light propagation in each coral compartment enabling predictions of, e.g., light exposure on the level of individual *Symbiodinium* cells inside the coral tissue under different regimes of incident solar radiation. Quantifying coral optical properties thus has important implications for understanding the ecophysiology of *Symbiodinium in hospite*.

The extraction of coral tissue optics is a difficult task due to the high optical density and the strong coupling of absorption and multiple scattering in coral tissue. However, suitable experimental and theoretical approaches have been developed in biomedical optics for non-invasive optical characterisation of complex tissues (Tuchin, 2007; Welch and van Gemert, 2011) driven by the need for early detection of tissue abnormalities and the design of efficient radiative treatment of cancer. A key approach involves the use of optical reflection spectroscopy in combination with radiative transfer modeling of the experimental data with Monte Carlo simulations calculating the photon probability distribution within biological tissues (Wang et al., 1995).

The aim of this paper is to provide estimates of the inherent optical properties of coral tissue. We use a well-established method developed in medical tissue optics and apply it to the study of coral optical properties. Specifically, we develop a 3D Monte Carlo simulation that is based on the widely used Monte Carlo code of photon propagation for multilayered tissues (Wang et al., 1995). We present an estimation of the inherent optical properties of intact faviid coral tissues that enables a simulation of light propagation through a simplified coral skeleton and tissue model. The present study provides a more detailed mechanistic understanding of the recent observations on light propagation in massive thick-tissued corals (Wangpraseurt et al., 2012, 2014a,b) and has important implications for the ecophysiology of corals and Symbiodinium photosynthesis *in vivo*.

## Materials and Methods

### Monte Carlo Model to Extract Coral Optical Properties

#### Theory

Monte Carlo simulations are a well-established and versatile approach toward modeling light propagation in tissues. Light transport in biological tissues is described by the radiative transfer equation (RTE), which simplifies Maxwell’s equations as it does not include non-linear properties of light such as interference, polarization and diffraction (Welch and van Gemert, 2011). Yet, an exact analytical solution to the RTE is difficult to obtain, given the complexity of biological tissues. Monte Carlo simulations have been widely used in medical tissue optics to provide a numerical solution to the RTE (Tuchin, 2007) by employing probability distribution models, where a photon propagates through a tissue with independent absorption and scattering centers (Wang et al., 1995). Numerous photons are launched and interact with the tissue as a random process of absorption or scattering. However, the overall probability of absorption and scattering are based on the optical parameters of the tissue (Wang et al., 1995). Through random interaction with a large set of photons, a characteristic light distribution is obtained that is governed by the optical parameters and the tissue geometry outlined in the model. Monte Carlo Simulations provide high accuracy even in strongly light absorbing media and have the advantage that in principle any source geometry can be modeled. The disadvantage is that they are computationally and time expensive given that a large number of photons need to be simulated (typically >1 × 10^6^ photons).

Monte Carlo methods are often used in combination with diffuse reflectance spectroscopy (e.g., Farrell et al., 1992; Wang and Jacques, 1993) to solve the inverse problem of the RTE, i.e., to predict the inherent optical properties of a tissue based on measured light field parameters. This typically involves measuring the lateral attenuation of the fluence rate (also called scalar irradiance) for several light source and light detector distances. Monte Carlo Simulations are then used to extract a unique set of optical properties that generates the measured lateral attenuation of the fluence rate. The present study uses the recently developed 3D Monte Carlo code mcxyz (Jacques et al., 2013) based on the multilayered tissue Monte Carlo algorithm (Wang et al., 1995) that is widely regarded as the gold standard for modeling photon propagation in tissues (Tuchin, 2007). The code developed by Wang et al. (1995) has been validated by numerous investigators using phantoms with known optical properties.

#### Optical Data for Monte Carlo Model

To understand the recent observation of lateral light transfer in faviid coral tissues and extract its inherent optical properties, the optical data generated in Wangpraseurt et al. (2014a) were used to feed the Monte Carlo simulation. Briefly, the optical data consisted of fine-scale measurements of the lateral spread of an incident laser beam on (i) the intact faviid coral and (ii) the bare skeleton. The *Favia speciosa* coral had brown/gray tissue which was about 2 mm thick as measured with microsensors (Wangpraseurt et al., 2014a). *F. speciosa* corals have on average moderate *Symbiodinium* cell densities amounting to about 2 × 10^6^ cells cm^-2^ surface area (Li et al., 2008). The basic dimensions of the individual skeleton corallites were measured with a ruler and determined to be on average about 12 mm in diameter and about 8 mm in depth and with ∼3 mm wide skeletal walls (coenosteum). These basic dimensions were similar to measurements on other *F. speciosa* skeletons from Australia (cf. Australian Institute of Marine Science 2013, AIMS Coral Fact Sheets – *F. speciosa*).

The optical data were acquired by illuminating coenosarc tissue areas of the coral with a ∼2 mm wide red laser diode beam (636 nm, 4.5 mW; Edmunds Optics, Barrington, NJ, USA). Measurements were conducted on the coenosarc, which had a more even topography and less contractile tissue as compared with polyp tissue, thus allowing repeated measurements and more accurate estimates of horizontal light transfer. The light source was a circular beam laser-diode module. The laser beam had an emission of 636 nm with a beam diameter of 2.05 mm, which delivered an irradiance of 4.5 mW (Edmunds Optics, Barrington, NJ, USA) with a Gaussian intensity spectral distribution. The output power of the laser source was thus brighter than under natural conditions, but the intensity dropped to only about 2% of the incident power, i.e., less than 0.1 mW at the first point of measurement. The laser source was very stable with a power stability of ±2% and a wavelength stability 0.2–0.3 nm per °C. During the laser measurements no other light was applied. The lateral beam spread across coral tissue was mapped with scalar irradiance microprobes with a spherical tip diameter of 80 μm and an isotropic light collection (Lassen et al., 1992; Rickelt et al., 2016) that quantified fluence rate.

The lateral light distribution was mapped along seven positions of increasing distance from the point of light delivery with the aid of a micromanipulator (Märtzhäuser GmbH, Wetzlar, Germany) to ensure accurate positioning of the fiber microprobe relative to the incident light spot. The fiber-optic microprobes and laser diode were each mounted on a different micromanipulator (Pyro-Science, Aachen, Germany, and Märtzhäuser, Wetzlar, Germany) and aligned parallel to each other perpendicularly to the coral surface. To extract the optical properties from the radial attenuation of the fluence rate, optical measurements were performed in direct contact with the tissue surface (Welch and van Gemert, 2011). The fiber-optic microprobes were accurately positioned at and in direct contact with the tissue surface by the aid of a dissecting microscope and the use of the automatic profiling function of the micromanipulator (see Wangpraseurt et al., 2012). After experiments on the live coral, the tissue was removed with an air gun and experiments were repeated with the bare skeleton of the sample under otherwise identical underwater conditions; measurement spots were carefully selected to be similar to areas on the living coral. The experimental measurements were recorded as measures of radially detected light relative to the 100% signal obtained when the sensor was directly within the beam (Wangpraseurt et al., 2014a). We refer to this relative measure as, M(*r*) [a.u.].

#### Development of a Two-Layered Coral Structure Used in the Monte Carlo Model

The anatomical structure of both the coral tissue and skeleton are complex as the tissue has several cell layers (e.g., gastroderm, epi-/endo-derm, mesoglea) and the skeleton has an intricate structure that includes voids and pronounced microstructural heterogeneity. The primary aim of the development of the Monte Carlo simulations was to obtain first estimates of the bulk light scattering properties of coral tissue to understand the average light transport through coral tissue across the coral surface (i.e., for several mm). Therefore, we treated the tissue as a homogenous turbid (i.e., light scattering) medium. To extract the optics of the tissue, our approach relies on estimates of the bulk scattering properties of the coral skeleton. However, although two previous studies (Teran et al., 2010; Marcelino et al., 2013) have estimated the scattering coefficient of the skeleton, this data cannot be easily compared and used in this study. Teran et al. (2010) used slices of skeleton and an integrating sphere while Marcelino et al. (2013) used low coherence enhanced backscattering spectroscopy to determine the reduced scattering coefficient for a range of coral skeletons for short photon pathlengths (<100 μm). As the latter measurements were aimed at light that superficially interacts with the skeleton, their estimates of the reduced scattering coefficient present an estimate of the scattering in the locally monitored aragonite structure.

In contrast, our aim was to estimate the bulk scattering properties of the skeleton, and our optical data is thus based on light measurements of up to 2 cm distance (vs. 100 μm in Marcelino et al., 2013) across the coral surface thus averaging the structural complexity of the skeleton and light transport through aragonite and skeleton voids, i.e., treating the skeleton as a homogenous turbid medium with average optical properties.

**FIGURE 1:**
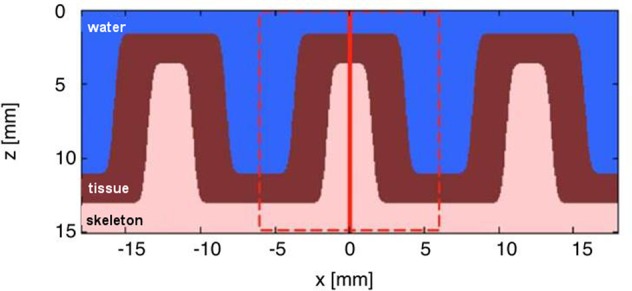
**Simplified structural 2-layer model of a faviid coral in *x, z* dimensions.** The model assumes the dimensions of a massive faviid coral with ∼3 mm thick skeletal walls (i.e., the coenosteum), ∼10 mm wide corallites and a ∼2 mm thick living tissue layer. The model extends uniformly along *y* and shows three layers: water, living tissue, and skeleton. The dashed red line indicates the region modeled by the Monte Carlo simulation. The vertical solid red line indicates the position of light delivery to the top of the skeleton/tissue wall.

The corallite structure was modeled using a 3D Monte Carlo simulation software routine, termed ‘mcxyz.c’ (Jacques et al., 2013), which uses the basic algorithm underlying the well-known MCML (Monte Carlo multi-layer) program (Wang et al., 1995). The structure was drawn in *x* (length) vs. *z* (depth) (as shown in **Figure 1**), and extended along the *y*-dimension (width) in Matlab (vs. 2014b; MathWorks Inc., Natick, MA, USA).

#### Extraction of Coral Skeleton Optical Properties from Monte Carlo Model

To solve the inverse radiative transfer problem for our two layer model, i.e., the extraction of inherent optical properties based on measured light distributions, we first estimated the bulk optical properties of the coral skeleton. The basic algorithm involved approximating starting values for μ_a_ and μ_s_ to run the first simulation. The initial Monte Carlo simulation of light attenuation across the skeleton surface was compared to the actual optical measurements. Based on this, a set of μ_s_’ and μ_a_ values were chosen, modeled and the predicted behavior of ϕ(*r*), i.e., the lateral attenuation of the fluence rate as a function of radial distance, compared to the optical measurements. These simulations thus created a look-up table that indicated the attenuation of the fluence rate, ϕ as a function of distance from the incident laser beam, r, for the different μ_s_’ and μ_a_ combinations. The different combinations are then compared and matched to the actual measurement through a non-linear least squares fitting algorithm. The non-linear least squares fitting algorithm is based on the fminsearch.m routine in MATLAB (Mathworks, Inc.), which uses a multidimensional unconstrained non-linear minimization algorithm (Nelder–Mead). The algorithm fits M(*r*) = K ϕ(*r*), where M(*r*) is the experimental measurement (see above) and K is the instrument calibration that is a constant value (in counts cm^-2^). The algorithm fits the shape of the curve, whose slope becomes increasingly negative as μ_s_’ is increased. The calibration constant K only affects the magnitude of the fit, not the shape (Tuchin, 2007).

The look-up table showed that varying μ_a_ from 0.01 to 0.316 to 0.56 cm^-1^ did not affect the model outcome (data not shown), thus the absorption of the skeleton matrix was extremely low and the attenuation was mainly due to scattering along the optical path and due to losses as photons escaped laterally (*x*-axis) out the sides of the polyp walls. Light loss was dominated by the leakage out of the skeleton, not by the absorption in the skeleton. In principle, one needs both the lateral attenuation of the fluence rate (ϕ vs. r) and the absolute value of φ to specify the two unknown optical properties (μ_a_, μ_s_’) using the Monte Carlo approach (Palmer and Ramanujam, 2006). However, because the model was not affected by skeletal absorption, the lowest simulated μ_a_ value (μ_a_ = 0.01) was assumed representative for the skeleton, which matched known μ_a_ values for a range of coral skeletons, i.e., μ_a_ = ∼0.01 cm^-1^ at 600–650 nm (Marcelino et al., 2013). The fit between experiment and simulation of the coral skeleton was then only based on the best choice of μ_s_’ of the skeleton, which was 3.4 cm^-1^. These optical properties of the skeleton were then used for the extraction of the optics of coral tissue (see below).

#### Extraction of Coral Tissue Optical Properties from Monte Carlo Model

The optical properties of the coral tissue were extracted by assigning the optical properties of the coral skeleton (μ_a_ = 0.01 cm^-1^, μ_s_’ = 3.4 cm^-1^) and adding the coral tissue layer on top of the skeleton in the Monte Carlo model (**Figure 1**). The optical properties of the coral tissue were varied over 5 μ_a_ values (0.01, 0.056, 0.0316, 1.78, and 10 cm^-1^) and 5 μ_s_’ values (0.1, 0.562, 3.15, 17.8, and 100 cm^-1^). The range of values was chosen based on known scattering and absorption properties of other biological tissues including human skin and plants (Ganapol et al., 1998; Jacques, 2013). The Monte Carlo simulations were matched to the measurements on the intact coral with a non-linear least squares fitting routine (as above).

#### Normalization and Calibration of the Data

**FIGURE 2:**
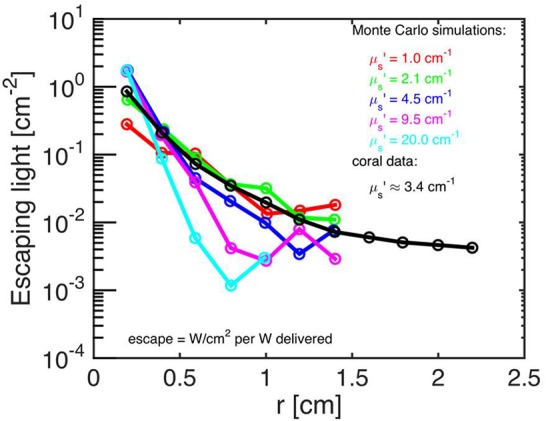
**Monte Carlo simulations to estimate coral skeletal optical properties.** Escaping light [ϕ; W/cm^-2^ per W delivered] as a function of radial distance (r) from the incident laser beam, as measured along the bare skeleton surface. Monte Carlo simulations were run for the 5 μ_s_’ values (colored numbers) and for a constant μ_a_ = 0.01 cm^-1^. The best fit of the experimental data (black) was μ_s_’ = 3.4 cm^-1^ (*n* = 15 repetitions).

Both the Monte Carlo and the experimental data were normalized by the values at *r* = 2 mm, i.e., ϕ(*r*)/ϕ(*r* = 2 mm) and M_skeleton_(*r*)/M_skeleton_(*r* = 2 mm). This normalization allowed comparison of the data, despite the differences in units between the experimental data (in relative units; a.u.) and the simulations (in absolute units; W cm^-2^). The experimental data, M(*r*) [a.u.], were then related to the simulated fluence rate values as: ϕ(*r*)_skeleton_ = KM_skeleton_(*r*)/M_skeleton_(2 mm), where K was the instrument calibration (in counts cm^-2^) determined through the Nelder–Mead non-linear minimization algorithm (see above). The calibration constant CALIB was specified as CALIB = K/M_skeleton_(2 mm), and the experimental data were calibrated as ϕ(*r*)_skeleton_ = CALIB M_skeleton_(*r*) (**Figure 2**).

#### Assumptions of the Model

The major unknown parameters in our model are the refractive index and the anisotropy of scattering, g, describing the relative forward propagation of light via scattering. The *g* value is difficult to determine for optically thick samples such as a multilayered coral tissue and the coral skeleton, but for most biological tissues the *g* value is close to but less than unity and light is strongly forward scattered (Jacques, 2013). Here, we used a value of *g* = 0.9, which is commonly used in tissue optics for light in the visible range (Jacques, 2013). The Monte Carlo simulations used the scattering coefficient μ_s_ and the assumed *g* value to calculate the reduced scattering coefficient μ_s_’ = μ_s_ × (1-*g*).

The 3D Monte Carlo code (Jacques, 2013) assumes that the refractive index (*n*) was matched at the surface boundary between coral tissue and seawater. While this is not exactly correct, the error was expected to be small. The n of coral is ∼1.38 (for ∼70% water content) and the n of seawater is ∼1.33, and the ratio 1.38/1.33 yields a total internal reflectance (r_i_) of 0.08 (Groenhuis et al., 1983). In contrast, a coral tissue exposed to air (*n* of air is 1) would yield a ratio of 1.38/1 and *r*_i_ = 0.51 (Groenhuis et al., 1983). This *r*_i_ quantifies the fraction of light attempting to escape the tissue that is internally reflected back into the tissue.

### Monte Carlo Simulation to Illustrate Photon Propagation through a Living Coral

Based on the extracted optical properties of the coral tissue and skeleton, we simulated photon propagation through an intact coral for different angles of incident irradiance. First, the light distribution was simulated for the case, where an incident collimated beam was uniformly delivered vertically from above (noon time direct sun). Next, the light distribution was simulated for the case where light was uniformly delivered at a 45° direct sun angle. Finally, the 45° angle simulation was repeated, but with the skeleton properties changed to equal the coral tissue properties, as if there was no skeleton but just coral tissue. This simulation was done to explore how tissue and skeleton optics affect light propagation.

## Results

### Extraction of Optical Parameters Based on Monte Carlo Simulations

**FIGURE 3:**
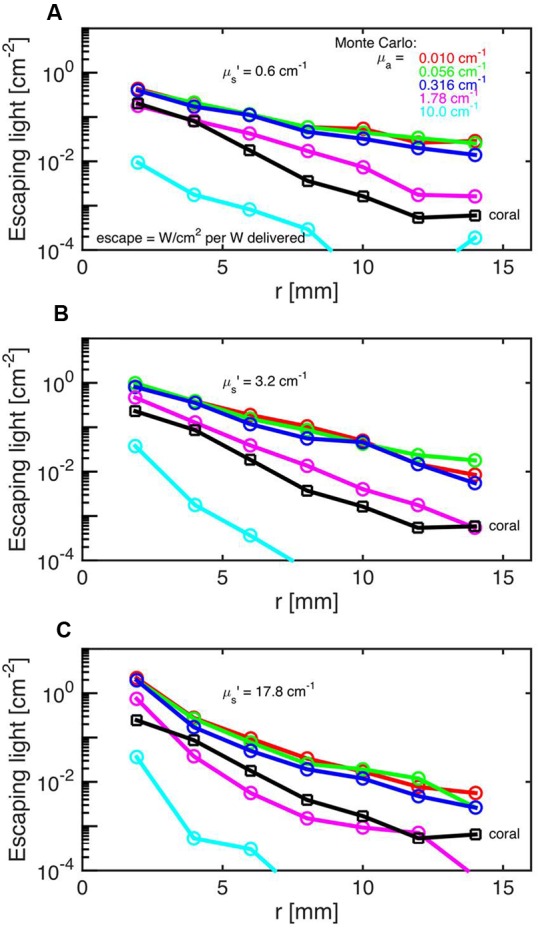
**Monte Carlo simulations to estimate coral tissue optical properties.** Escaping light (ϕ) as a function of radial distance (r) from the incident laser light beam as measured over the intact coral tissue surface. Monte Carlo simulations were run for 5 μ_a_ values, assuming μ_s_’ = 0.6 **(A)**, 3.2 **(B)**, and 17.8 cm^-1^ **(C)**. The experimental data are black squares (mean ± SD, *n* = 13 repetitions). The match between experiment and simulation was based on both the slope and absolute values of the ϕ(r) curves. The best choice was μ_a_ = 1.8 cm^-1^ and μ_s_’ = 10 cm^-1^.

Monte Carlo simulations showed that the investigated faviid coral skeleton exhibited low absorption and moderate light scattering, with optical properties of μ_a_ = 0.01 cm^-1^ and μ_s_’ = 3.4 cm^-1^ (at 636 nm; **Figure 2**). In contrast, the probability of absorption and scattering within the tissue was much higher, as indicated by a μ_a_ of 1.8 cm^-1^ and a μ_s_’ of 10 cm^-1^ at 636 nm (**Figures 3A–C**).

### Monte Carlo Simulation of Photon Propagation through Coral Tissue and Skeleton

**FIGURE 4:**
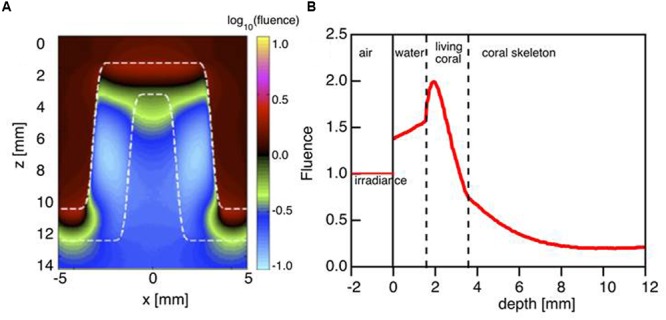
**Monte Carlo simulation of light distribution in a faviid coral for vertically incident collimated illumination. (A)** The 2D distribution of relative 636 nm fluence rate ϕ (*z,x*) in the model coral (as in **Figure 1A**). **(B)** The axial profile down the center of the coral coenosteum ϕ (*z*) at *x* = 0 mm. Note that the fluence rate within the living tissue is twice the delivered irradiance (1 cm^-2^). Between *z* = 0 to 2 mm, the back-reflected light escaping from the tissue adds to the delivered irradiance to yield a fluence rate in water that exceeds 1 cm^-2^.

Based on the coral optical properties obtained from the Monte Carlo simulations, the relative fluence rate was modeled at 636 nm within the tissue during mid-day under vertically incident sunlight. This showed that the fluence rate (ϕ) within the coral tissue can reach up to twice the incident irradiance at a depth of 50–100 μm (*E* = 1 W cm^-2^ per W delivered; **Figures 4A,B**). Note that the escaping flux from the coral adds to the incident light to cause the fluence rate to be enhanced by up to 1.5 times in the water above the tissue (**Figure 4B**).

**FIGURE 5:**
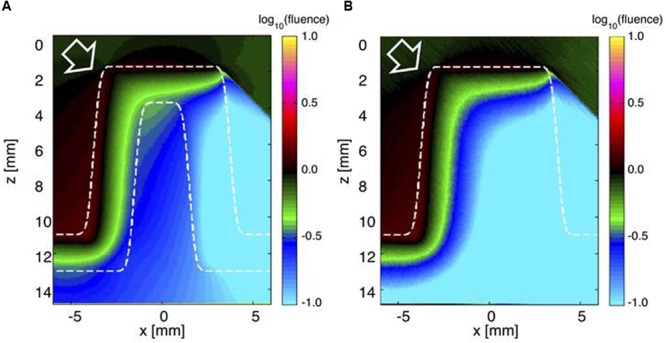
**Monte Carlo simulation of light distribution in a faviid coral under oblique incident illumination.** The white arrow indicates the direction of incident collimated light (at 45°). **(A)** The 2D distribution of relative 636 nm fluence rate ϕ (*z,x*) across the model coral (tissue and skeleton). **(B)** The ϕ (*z,x*) image if the skeleton was replaced by living tissue.

To illustrate the role of the skeleton in light propagation, light was delivered obliquely at a 45° angle to the living coral with tissue and skeleton (**Figure 5A**) and to a hypothetical coral structure, where the skeleton was replaced by a solid mass of living tissue (**Figure 5B**). In the living coral scenario, light penetrated the tissue and skeleton to reach the opposite side of the corallite (**Figure 5A**). However, without the skeleton light penetration to the other side was strongly reduced. The optical properties of the skeleton thus apparently enabled low but still significant amounts of light to reach otherwise shaded parts of coral tissue.

## Discussion

We present the first estimates of the inherent optical properties of coral tissue. By using a combination of radiative transfer modeling and optical data, we calculated a high reduced scattering coefficient of μ_s_’ = 10 cm^-1^ at 636 nm for the tissue of a thick-tissued massive coral. The reduced scattering coefficient of the coral skeleton was about three times lower (μ_s_’ = 3.4 cm^-1^ at 636 nm).

These findings support earlier suggestions regarding the strong tissue light scattering properties of faviid corals and their role in modulating coral light fields (Kühl et al., 1995; Wangpraseurt et al., 2012, 2014a,b; Brodersen et al., 2014). The high reduced scattering coefficient of coral tissue is comparable to the scattering of plant leaves (Ganapol et al., 1998) and human tissues such as muscle (μ_s_’ = 9 cm^-1^) (Patterson et al., 1989). Simulations of vertical light propagation (636 nm) through a coral polyp predicted fluence rate maxima reaching about two times the incident just below the tissue surface (**Figure 4B**). This suggests that coral tissues can employ similar optical mechanisms as plant leaves, where strong scattering of light within the plant tissue enhances fluence rate and maximizes photosynthesis (Seyfried and Fukshansky, 1983; Vogelmann, 1993). Intense scattering in the upper tissue layers is followed by rapid light attenuation through the tissue and thus the presence of pronounced vertical light gradients. These findings from a simple radiative transfer model are in good agreement with actual *in vivo* measurements of tissue fluence rate gradients in other massive corals (Wangpraseurt et al., 2012, 2014b).

As incident light reaches the skeleton, the model predicts that light attenuation is strongly reduced relative to the tissue (**Figures 4** and **5**). The low reduced scattering and absorption coefficients of the skeleton could thus facilitate efficient light propagation through the skeletal matrix **Figure 4B.** Our Monte Carlo simulations exemplify the benefit of such skeletal light propagation, when solar radiation is obliquely incident, as it would occur on a coral reef during mornings or afternoons. Light can thus travel across polyp walls, i.e., through the corallite skeleton and illuminate tissue areas not directly exposed by the sun leading to an overall more homogeneous light exposure of the coral.

Our estimates of coral skeleton μ_s_’ values suggest that the 3-D skeleton structure of a massive faviid coral can be compared to other low scattering structures such as bone (∼μ_s_’ = 3.5 cm^-1^; Firbank et al., 1993). Notably, the μ_s_’ estimates reported here are one order of magnitude lower than the average values reported for dried coral skeletons in Marcelino et al. (2013), while our estimates of coral skeleton μ_a_ values agree well with previous findings suggesting that the visible light absorption of the skeleton is fairly negligible (Teran et al., 2010; Marcelino et al., 2013). Visible light absorption is much higher in the pigmented coral tissue, where the irradiance and spectral distribution is affected by characteristic photopigments present in coral microalgae (Stambler and Dubinsky, 2005; Hochberg et al., 2006) as well as fluorescent and non-fluorescent host pigments in the coral tissue (Salih et al., 2000; Lyndby et al., 2016).

The extraction of the inherent optical properties of coral tissue bears several implications for understanding the optical microenvironment and ecophysiology of *Symbiodinium in hospite*. Firstly, for the investigated massive faviid corals it shows that tissue scattering is an important optical mechanism enhancing the local irradiance regime available for symbionts in the upper tissue layers (**Figures 4A,B**). The high values of photosynthetic quantum efficiencies (∼0.1 O_2_ photon^-1^ (Brodersen et al., 2014) found in upper tissue layers of massive corals are thus affected by the extent to which tissue scatters light.

At the same time, intense tissue light scattering leads to a rapid loss in irradiance facilitating the presence of moderate light environments for symbionts close to the skeleton. We have recently used nanoscale secondary ion mass spectroscopy to identify carbon fixation rates of individual *Symbiodinium* cells within the tissue of faviid corals and found that although only little light penetrated toward tissue layers close the skeleton, such low levels of irradiance were efficiently used by the remaining *Symbiodinium* cells that appeared to be adapted to the low levels of irradiance (Wangpraseurt et al., 2016). It was further shown that under excess levels of incident irradiance *Symbiodinium* in aboral tissue layers were able to photosynthesise efficiently compared to *Symbiodinium* in oral tissue layers, which were strongly photoinhibited under the same levels of incident irradiance (Lichtenberg et al., 2016). Thus, the optical properties of a faviid coral leads to light gradients and optical shelter for *Symbiodinium* cells in aboral tissue layers, which can be especially useful during periods of excess radiation.

**FIGURE 6:**
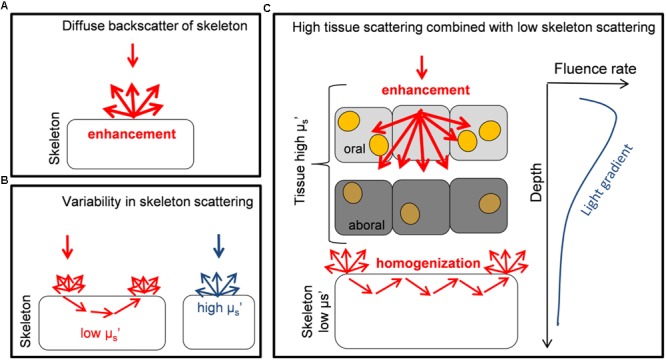
**Schematic diagram illustrating different optical strategies of corals. (A)** The diffuse backscattering properties of skeletons from the coral *Porites branneri* have been measured and it was shown that the skeleton was highly reflective with an almost isotropic distribution of the backscattered light. Such scattering can lead to a diffusely enhanced light field for *Symbiodinium* (based on Enriquez et al., 2005). **(B)** Low-coherence enhanced backscattering spectroscopy has been used to identify that the optical properties of coral skeletons show a great variability in the microscopic reduced scattering coefficient μ_s_’ (for short photon pathlengths). It is shown that a continuum of skeletal scattering properties exist, which are affected by the fractal dimensions of the skeleton (based on Marcelino et al., 2013). **(C)** Monte Carlo simulations are used to identify the optical properties of live faviid corals including intact coral tissue. A combination of light transport is identified where the tissue strongly scatters and enhances irradiance locally, while the skeleton homogenizes and distributes light to otherwise shaded areas. These two optical strategies are used to counteract the light gradient present in coral tissues. *Symbiodinium* cells (yellow dots) in oral tissue layers receive high amounts of light while in aboral tissue layers irradiance is reduced and *Symbiodinium* (dark orange) uses the remaining low light efficiently (based on this study and Wangpraseurt et al., 2012, 2014a, 2016).

The optical strategy of a faviid coral identified here is different to the light harvesting strategies identified previously for other corals (**Figure 6**). In contrast to coral species for which strong skeletal backscattering creates evenly enhanced *in hospite* irradiance (as in a thin-tissued branching coral, Enriquez et al., 2005; Teran et al., 2010; Marcelino et al., 2013), the optical properties of fleshy faviid corals allow for substantial heterogeneities in the optical microenvironment even within the 2 mm thin coral tissue (Wangpraseurt et al., 2012; **Figure 6**). It is very likely though that there is a much greater diversity of optical strategies in corals as currently identified (**Figure 6**), given that the structure and biochemical composition of coral tissues and skeletons are highly diverse (Perrin, 2003; Marcelino et al., 2013). It has been suggested that skeletal scattering differs along a continuum between different coral species (Marcelino et al., 2013) and as a function of water depth, where the diffuse reflectance of skeletons increases in deeper waters (Kahng et al., 2012). Likewise, using optical coherence tomography our ongoing studies suggest that the scattering coefficient of coral tissue differs between species (D. Wangpraseurt, unpublished data). There is also evidence that green-fluorescent protein-like pigments are important components of tissue light scattering (Lyndby et al., 2016). The density of such GFP granules differs between species and tissue types. For instance, GFP granules are characteristic for polyp tissues of some thick-tissued faviid corals while they are less prevalent for other coral species (e.g., brown *Pocillopora damicornis*). Thus it appears that there is a great diversity of light harvesting strategies on coral reefs that, we are just beginning to understand.

Radiative transfer modeling by means of Monte Carlo simulations is a powerful tool for studying the biophotonics of corals. Future studies should aim at qualifying the simplifications, we have made here regarding the structural complexity of both tissue and skeleton, e.g., by employing non-invasive imaging techniques, such as optical coherence tomography that can gain high-resolution data of tissue structure and scattering properties (Levitz et al., 2004). More complex heterogeneous surfaces could then be modeled in a 3D Monte Carlo simulation in order to improve upon the structural simplifications, we have made in this first study. In general, a heterogeneous surface topography can lead to multiple scattering at the tissue surface and subsurface (Bennett and Porteus, 1961; He et al., 1991). It is thus likely that surface roughness would further affect our fluence rate estimates in the uppermost tissue depths. Our Monte Carlo simulations assumed a simplified skeletal structure, i.e., without the intricate coral skeleton architecture with different skeleton densities and voids. The general tendency is that such voids reduce the μ_s_’ (Marcelino et al., 2013), which would further support the efficient light propagation of skeletons proposed here.

## Conclusion

We have provided an estimate of the inherent optical properties of intact massive faviid corals based on radiative transfer modeling of experimental data. Our results reveal an interesting interplay between skeleton and tissue optics in optimizing coral light harvesting and suggest two different ‘modes’ of photon propagation in the two coral compartments: Light enhancement and high scattering in the coral tissue as characterized by a high μ_s_’ vs. light field homogenization and low scattering in the coral skeleton as indicated by a much lower μ_s_’. Overall, these optical properties likely facilitate efficient photosynthesis in the coral tissue of massive faviid corals.

## Author Contributions

DW, SJ, and MK designed research; DW performed research; DW and SJ developed model. SJ, TP, and MK contributed new reagents/analytic tools; DW, SJ, TP, and MK analyzed data; and DW, SJ, and MK wrote the paper.

## Conflict of Interest Statement

The authors declare that the research was conducted in the absence of any commercial or financial relationships that could be construed as a potential conflict of interest.
